# Behavior of blood plasma glycan features in bladder cancer

**DOI:** 10.1371/journal.pone.0201208

**Published:** 2018-07-24

**Authors:** Shadi Ferdosi, Thai H. Ho, Erik P. Castle, Melissa L. Stanton, Chad R. Borges

**Affiliations:** 1 School of Molecular Sciences, Arizona State University, Tempe, AZ, United States of America; 2 Virginia G. Piper Center for Personalized Diagnostics, The Biodesign Institute at Arizona State University, Tempe, AZ, United States of America; 3 Division of Hematology and Medical Oncology, Mayo Clinic, Phoenix, AZ, United States of America; 4 Department of Urology, Mayo Clinic, Phoenix, AZ, United States of America; 5 Department of Laboratory Medicine and Pathology, Mayo Clinic, Phoenix, AZ, United States of America; University of South Alabama Mitchell Cancer Institute, UNITED STATES

## Abstract

Despite systemic therapy and cystectomy, bladder cancer is characterized by a high recurrence rate. Serum glycomics represents a promising source of prognostic markers for monitoring patients. Our approach, which we refer to as “glycan node analysis”, constitutes the first example of molecularly “bottom-up” glycomics. It is based on a global glycan methylation analysis procedure that is applied to whole blood plasma/serum. The approach detects and quantifies partially methylated alditol acetates arising from unique glycan features such as α2–6 sialylation, β1–4 branching, and core fucosylation that have been pooled together from across all intact glycans within a sample into a single GC-MS chromatographic peak. We applied this method to 122 plasma samples from former and current bladder cancer patients (n = 72 former cancer patients with currently no evidence of disease (NED); n = 38 non-muscle invasive bladder cancer (NMIBC) patients; and n = 12 muscle invasive bladder cancer (MIBC) patients) along with plasma from 30 certifiably healthy living kidney donors. Markers for α2–6 sialylation, β1–4 branching, β1–6 branching, and outer-arm fucosylation were able to separate current and former (NED) cases from certifiably healthy controls (ROC curve c-statistics ~ 0.80); but NED, NMIBC, and MIBC were not distinguished from one another. Based on the unexpectedly high levels of these glycan nodes in the NED patients, we hypothesized that recurrence of this disease could be predicted by some of the elevated glycan features. Indeed, α2–6 sialylation and β1–6 branching were able to predict recurrence from the NED state using a Cox proportional hazards regression model adjusted for age, gender, and time from cancer. The levels of these two glycan features were correlated to C-reactive protein concentration, an inflammation marker and known prognostic indicator for bladder cancer, further strengthening the link between inflammation and abnormal plasma protein glycosylation.

## Introduction

Urothelial cell carcinoma (UCC) or bladder cancer is one of the top ten causes of cancer deaths annually [1]. From a clinical perspective, there are two major forms of this cancer: 1) non-muscle-invasive bladder cancer (NMIBC; stages pTa/pT1/pTis) and 2) muscle-invasive bladder cancer (MIBC; stages pT2+). Early detection of bladder cancer is very important; patients with non-muscle-invasive tumors have a much higher 5-year survival rate—88% for NMIBC patients relative to 41% for MIBC patients [2]. Yet despite the stage at which it is diagnosed, high recurrence rate is one of the essential characteristics of this cancer [3]. Therefore, even if diagnosed at early stages and treated, former bladder cancer patients need to be monitored frequently. Currently, common methods for detecting bladder cancer and monitoring for its recurrence include: cystoscopy (which is invasive and expensive [4]), urine cytology (which has low sensitivity for low-grade bladder cancer [5]), and computed tomography (CT) screening (which may not detect small tumors [6]). Accordingly, there has been a wide search for new biomarkers that are noninvasive, cost effective, and can outperform cytology [7–10].

At present there are no clinically employed serum-based markers for monitoring patients after their treatment. Targeted glycomics, particularly when combined with other well defined markers and risk stratification models, represents a promising source for a new generation of bladder cancer markers [11]. Some evidence toward this end based on the detection of the Sialyl Lewis^a^ antigen [12, 13] and analysis of intact N-glycans [14, 15] in blood plasma/serum (P/S) from bladder cancer patients has been obtained. Aberrant glycosylation is a universal feature of cancer [16] where it appears to enable the ability of tumor cells to avoid innate immune detection [17]. The changes in structure and abundance of glycans are often caused by dysregulated glycosyltransferase (GT) activity [16]. Thus conceptually, a targeted glycan analysis technique that could provide one-to-one surrogate data for abnormal GT activity using routinely available clinical samples and that relied upon existing clinical technology could be quite valuable.

**Fig 1:**
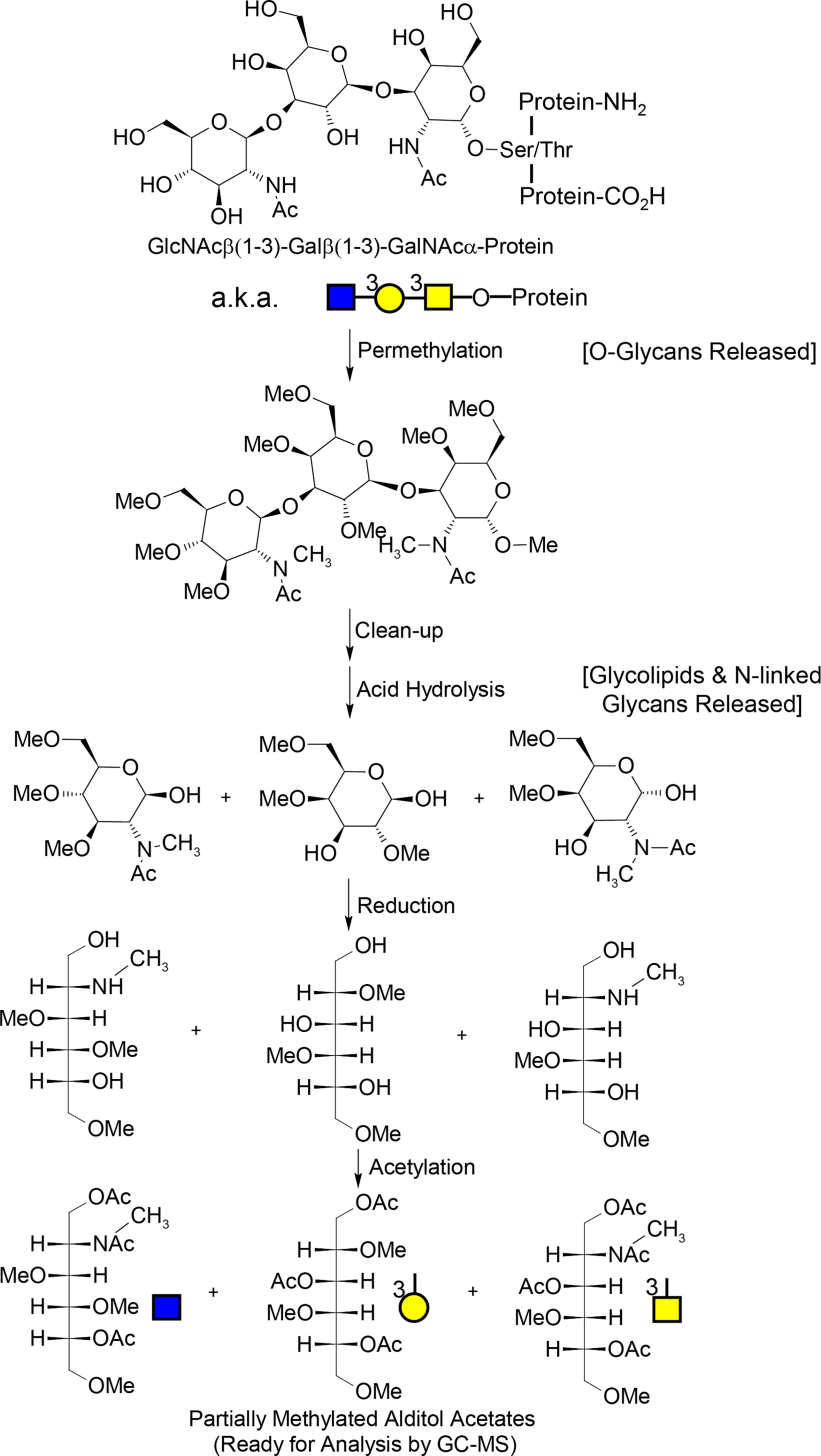
Molecular overview of the glycan “node” analysis procedure. For glycans from blood plasma and other biofluids, O-linked glycans are released during permethylation, while N-linked glycans and glycolipids are released during acid hydrolysis. The unique pattern of methylation and acetylation in the final partially methylated alditol acetates (PMAAs) corresponds to the unique “glycan node” in the original glycan polymer and provides the molecular basis for separation and quantification by GC-MS. Figure adapted with permission from Borges CR et al. Anal. Chem. 2013, 85(5):2927–2936. Copyright 2013 American Chemical Society.

**Fig 2:**
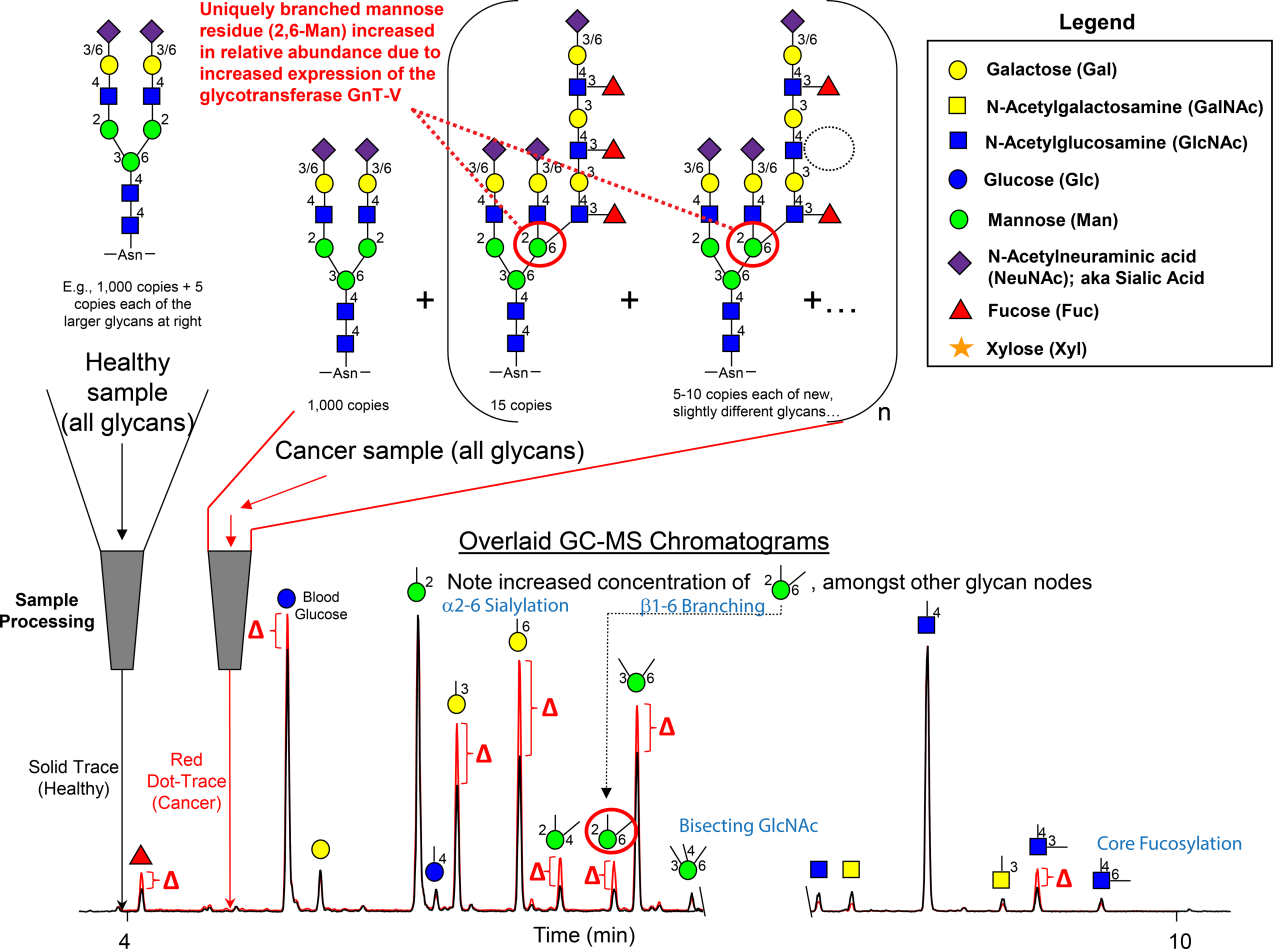
Conceptual overview of the glycan “node” analysis concept. The procedure consists of applying glycan methylation analysis (i.e., linkage analysis) to whole biofluids. Intact normal and abnormal glycans including O-glycans, N-glycans and glycolipids, are processed and transformed into partially methylated alditol acetates (PMAAs, Fig 1), each of which corresponds to a particular monosaccharide-and-linkage-specific glycan “node” in the original polymer. As illustrated, analytically pooling together the glycan nodes from amongst all the aberrant intact glycan structures provides a more direct surrogate measurement of abnormal glycosyltransferase activity than any individual intact glycan, while simultaneously converting unique glycan features such as “core fucosylation”, “α2–6 sialylation”, “bisecting GlcNAc”, and “β1–6 branching” into single analytical signals. Actual extracted ion chromatograms from 9-μL blood plasma samples are shown. Numbers adjacent to monosaccharide residues in glycan structures indicate the position at which the higher residue is linked to the lower residue. Figure adapted with permission from Borges CR et al. Anal. Chem. 2013, 85(5):2927–2936. Copyright 2013 American Chemical Society.

In 2013, we developed a molecularly bottom-up approach called glycan node analysis that, unlike other approaches used in P/S glycomics, focuses on the analysis of monosaccharide and linkage-specific glycan “nodes” instead of intact glycans [18–21]. It does this by employing the principles and processing chemistry of glycan methylation analysis (i.e., linkage analysis; Fig 1) to unfractionated P/S. This pools together each unique monosaccharide-and-linkage-specific glycan feature or glycan “node” from across all the normal and aberrant glycan structures in a given sample, providing a more direct surrogate measurement of GT activity than any single intact glycan. Moreover, many of these glycan nodes correspond directly and quantitatively to interesting glycan features such as “core fucosylation”, “bisecting GlcNAc”, and “β1–6 branching”—all captured as single GC-MS chromatographic peaks (Fig 2)

Our recent large lung cancer study provided important information about the diagnostic and prognostic value of P/S glycan nodes in lung cancer as well as other types of cancer [21]. In particular, we observed strong stage-dependence, but tissue-of-tumor-origin independence of elevated P/S glycan features. Moreover, we found that glycan nodes corresponding to α2–6 sialylation, β1–4 branching and β1–6 branching were able to predict survival and progression. The primary purposes of this study were to evaluate the ability of unique glycan features, quantified via glycan node analysis, to 1) evaluate the potential ability of glycan nodes to distinguish MIBC from NMIBC, 2) distinguish NMIBC patients from patients with a history of bladder cancer but currently exhibiting no clinical evidence of disease (NED), and 3) evaluate the ability of glycan nodes to predict recurrence from a state of remission (i.e., the NED state). Based on our observations in lung cancer [21], we anticipated findings of potential clinical interest under each objective. Moreover, elevated blood plasma protein glycosylation is known to be associated with inflammation in some non-cancerous clinical conditions [22–24]. Since C-reactive protein (CRP) is a well-studied marker of inflammation [25] as well as a prognostic marker for UCC [26–29], we also evaluated the quantitative relationship between glycan nodes that were prognostically useful in NED patients and CRP.

## Materials and methods

### Plasma samples

EDTA plasma samples from MIBC (n = 12), NMIBC (n = 39) and NED patients (n = 72), as well as certifiably healthy living kidney donors (n = 30) were enrolled in the Multidisciplinary Biobank at Mayo Clinic Arizona under a Mayo Clinic Institutional Review Board (IRB)-approved protocol. Patients eligible for enrollment were those seen at Mayo Clinic Arizona who were ≥ 18 years old, able to provide informed consent, and undergoing evaluation as either a potential living kidney donor or for genitourinary diseases. Detailed inclusion & exclusion criteria for living kidney donors are provided in Supporting Information (S1 Appendix). None of the living kidney donor patients smoked at the time of health screening and blood collection; 27% were former smokers and 73% never smoked. Living kidney donor and UCC patients were excluded if they declined to participate or if the banking of their biospecimens would compromise the availability of tissue for diagnosis and standard clinical care. All specimens were collected during the time frame of June 2010 through Feb. 2016. Standard operating protocols and blood collections were performed as previously described [30]. All specimens were stored at -80°C prior to shipment to ASU and maintained at -80°C at ASU prior to analysis. All specimens were analyzed blind and in random order. An aliquot of plasma from the same individual donor was analyzed in every batch as a quality control (QC) specimen to ensure batch-to-batch consistency.

This research was approved by Arizona State University’s IRB and all clinical investigations were conducted according to the principles expressed in the Declaration of Helsinki.

### Glycan node analysis

#### Sample preparation

Glycan node analysis was performed on the plasma samples as described previously [19]. Briefly, it includes four main steps (Fig 1): 1) permethylation, in which 9μL of plasma sample containing 1μL of a 10 mM solution of heavy-labeled D-glucose (U-^13^C_6_, 99%; 1,2,3,4,5,6,6-D_7_, 97%−98%) (Cambridge Isotope Laboratories), and N-acetyl-D-[UL-^13^C_6_]glucosamine (Omicron Biochemicals, Inc.) as the internal standard was mixed with 270μL of dimethylsulfoxide (DMSO) (Sigma-Aldrich) followed by 105 μL of iodomethane (99%, Cat. No. I8507, Sigma-Aldrich). Then, this mixture was added to a plugged 1-mL spin column (ThermoFisher Scientific, Waltham, MA, Cat. No. 69705) containing ~0.7g of sodium hydroxide beads (20–40 mesh, Sigma-Aldrich) which had been preconditioned by acetonitrile (Fisher Scientific) and washed twice with DMSO prior to addition of sample. After occasionally stirring the NaOH column over 11 min, the unplugged samples were spun for 15 s at 5,000 rpm (2,400g) in a microcentrifuge to extract the permethylated glycans. In order to maximize glycan recovery, 300μL of acetonitrile was added to the NaOH column and spun down for 30 s at 10,000 rpm (9,600g). Then, in a silanized 13 × 100 glass test tube holding 3.5 mL of 0.2 M sodium phosphate buffer, the solution from the first spin-through was added and mixed well. After pooling and mixing the second acetonitrile-based spin-through solution was combined with the rest of the sample, followed by 1.2 mL of chloroform (Sigma-Aldrich). The test tube was then capped and shaken well, followed by removal and discard of the aqueous layer. After two additional rounds of liquid/liquid extraction, the chloroform layer was recovered and dried under nitrogen at 74°C. 2) The second major step was TFA hydrolysis, in which 325 μL of 2M trifluoroacetic acid (TFA) (Sigma-Aldrich) was added to each test tube. After capping the samples and incubating them at 121°C for 2h, they were dried down under nitrogen at 74°C. 3) The third major step involved reduction of sugar aldehydes, in which the samples were incubated for an hour after adding 475 μL of a freshly made 10 mg/mL solution of sodium borohydride (Sigma-Aldrich) in 1M ammonium hydroxide (Sigma-Aldrich). Then 63 μL of methanol (Honeywell Burdick & Jackson) was added to each sample before drying at 74°C under nitrogen. Next, 125 μL of a 9:1 (v/v) methanol: acetic acid solution was added to each test tube followed by drying under nitrogen. To fully dry the samples, they were then placed in vacuum desiccator for approximately 20 min. 4) The fourth major step consisted of acetylation of nascent hydroxyl groups, in which the sample residue in each test tube was dissolved by 18 μL water before adding 250 μL of acetic anhydride (Sigma-Aldrich). After sonicating the samples for 2 min and incubating for 10 min at 60°C, 230 μL of concentrated TFA was added to each sample, followed by incubation of the capped samples for 10 min at 60°C. Then, 2 mL methylene chloride (Fisher Scientific) was added to each sample followed by 2 mL of water. Next, liquid/liquid extraction was done twice in which the methylene chloride layer was saved and then transferred into a silanized autosampler (ThermoFisher Scientific), dried under nitrogen, reconstituted in 120 μL of acetone (Avantor Performance Materials), and capped to be injected onto the GC-MS.

#### Gas chromatography-mass spectrometry

As previously described [21], an Agilent Model A7890 gas chromatograph (equipped with a CTC PAL autosampler) was used coupled to a Waters GCT (time-of-flight) mass spectrometer to analyze the prepared samples. For all samples, one injection of 1μL was made at split ratio of 20:1 onto an Agilent split-mode liner containing a small plug of silanized glass wool with the temperature set to 280°C. The DB-5ms GC column that was used for chromatography was 30 m. The oven temperature, initially kept at 165°C, was increased at a rate of 10°C/min up to 265°C. Immediately after that, the temperature was increased at a rate of 30°C/min to 325°C, then held constant for 3 min. The transfer line to the mass spectrometer was kept at 250°C. Following the elution of sample components from the GC column, they were subjected to electron ionization (70 eV, 250°C) and analyzed in the *m/z* range of 40–800 with a scan cycle time of 0.1 s. Daily calibration and tuning of the mass spectrometer was done using perfluorotributylamine.

The quantification method is described in detail elsewhere [18]. Briefly, summed extracted ion chromatogram peaks were integrated automatically and checked manually using QuanLynx software. The collected data were then exported to a spreadsheet for detailed analysis.

### Human C-reactive protein ELISA assay

The Invitrogen™ Human C-Reactive Protein ELISA kit (Catalog Number KHA0031, ThermoFisher Scientific) was used, following the manufacturer instructions, to measure the concentration of CRP in patient plasma samples. Final absorbance values were read at 450 nm by Thermo Scientific Multiskan Go plate reader and the concentration of samples were calculated using SkanIt Software 3.2.

### Statistical analysis

Individual extracted-ion chromatographic peak areas for each glycan node were normalized using one of two possible approaches: 1) Individual hexose residues were normalized to heavy glucose and individual N-acetylhexosamine (HexNAc) residues were normalized to heavy N-acetyl glucosamine (heavy GlcNAc). 2) Individual hexose residues were normalized to the sum of all endogenous hexose residues. Likewise, each HexNAc residue was normalized to the sum of all endogenous HexNAcs. The average %CV calculated based on the analysis of the QC sample in each batch shows that the latter normalization method provides better inter-batch reproducibility (< 10% for the four most elevated glycan nodes) but the former normalization method performs better in separating the patient groups while still keeping the average inter-batch %CV in an acceptable range (i.e., < 18%). Unless otherwise noted, results described below are based on normalization with heavy glucose and heavy GlcNAc. All extracted-ion chromatographic peak areas for all samples, including their normalization to heavy glucose or heavy GlcNAc and normalization to the sum of all endogenous hexoses or HexNAcs as well as %CV values for batch-to-batch QC samples are included in Supporting Information (S1 File).

For both the glycan node data and the CRP ELISA data, outliers within each clinical group (Control, NED, NMIBC and MIBC) were removed after log_10_ transformation using the ROUT method at Q = 1% by GraphPad Prism 7. After removing the outliers, the anti-log of each value was taken to reverse the transformation. To identify differences between clinical groups, the Kruskal-Wallis test was performed followed by the Benjamini-Hochberg false discovery correction procedure at a 5% false discovery rate using RStudio Version 1.0.143. Univariate distributions and ROC curves were plotted using GraphPad Prism 7. The ability of certain glycan nodes to predict bladder cancer recurrence was evaluated by performing Cox proportional hazards regression models using SAS 9.4. Correlations between CRP and glycan nodes were examined using Pearson correlation in GraphPad Prism 7.

## Results

### Altered glycan features in UCC

The relative abundance of 19 glycan “nodes” was quantified in each of the control, NED, NMIBC, and MIBC patient samples. Each of these nodes contributed at least 1% of the sum total of all hexoses or all HexNAcs. Data normalized to heavy, stable isotope-labeled glucose and GlcNAc internal standards were first evaluated for statistically significant differences between all four patient groups. No differences were found between MIBC, NMIBC and NED patients (**Table 1**). However, relative to the certifiably healthy controls, statistically significant changes were found in more than half of the glycan nodes measured in NED, NMIBC, and MIBC patients (**Table 1**). Among these glycan nodes, the only one that was *decreased* in the current and former cancer patient samples was 4-linked glucose (i.e., 4-Glc, which is mostly derived from glycolipids). The same trend was previously observed in lung cancer patient samples [21]. The rest of the altered nodes were *increased* in current and former UCC patients compared to the certifiably healthy controls.

**Table 1:** Statistically significant differences between controls and bladder cancer patient sub-cohorts^a^.

Glycan Nodes ^b^^,^^c^	Control vs. NED	Control vs. NMIBC	Control vs. MIBC	NED vs. NMIBC	NED vs. MIBC	NMIBC vs MIBC
**t-Fucose**	i	i	ii	ns	ns	ns
**t-Gal**	ns	ns	ns	ns	ns	ns
**2-Man**	iii	ii	iii	ns	ns	ns
**4-Glc**	ns	d	ns	ns	ns	ns
**3-Gal**	ns	ns	ns	ns	ns	ns
**6-Gal**	iiii	iii	ii	ns	ns	ns
**3,4-Gal**	ns	ns	ns	ns	ns	ns
**2,4-Man**	iii	ii	i	ns	ns	ns
**2,6-Man**	iiii	iiii	ii	ns	ns	ns
**3,6-Man**	i	ns	ns	ns	ns	ns
**3,6-Gal**	ns	ns	ns	ns	ns	ns
**3,4,6-Man**	i	i	i	ns	ns	ns
**t-GlcNAc**	i	i	ns	ns	ns	ns
**4-GlcNAc**	ii	ii	i	ns	ns	ns
**3-GlcNAc**	ii	i	ii	ns	ns	ns
**3-GalNAc**	ns	ns	i	ns	ns	ns
**3,4-GlcNAc**	ii	ii	ii	ns	ns	ns
**4,6-GlcNAc**	ns	ns	ns	ns	ns	ns
**3,6-GalNAc**	iii	ii	iii	ns	ns	ns

^a^ Individual hexose residues were normalized to heavy glucose and individual HexNAc residues were normalized to heavy GlcNAc).

^b^ Significance was determined by the Kruskal-Wallis test followed by the Benjamini-Hochberg correction procedure at a 5% false discovery rate.

^c^ “ns” stands for “not significant”. “i” and “d” stand for “increased” or “decreased” glycan levels in the cohort with clinically more advanced disease listed in the column header. “i” or “d” indicates *p* < 0.05, “ii” or “dd” indicates *p* < 0.01, “iii” or “ddd” indicates *p* < 0.001, and “iiii” or “dddd” indicates *p* < 0.0001.

**Fig 3:**
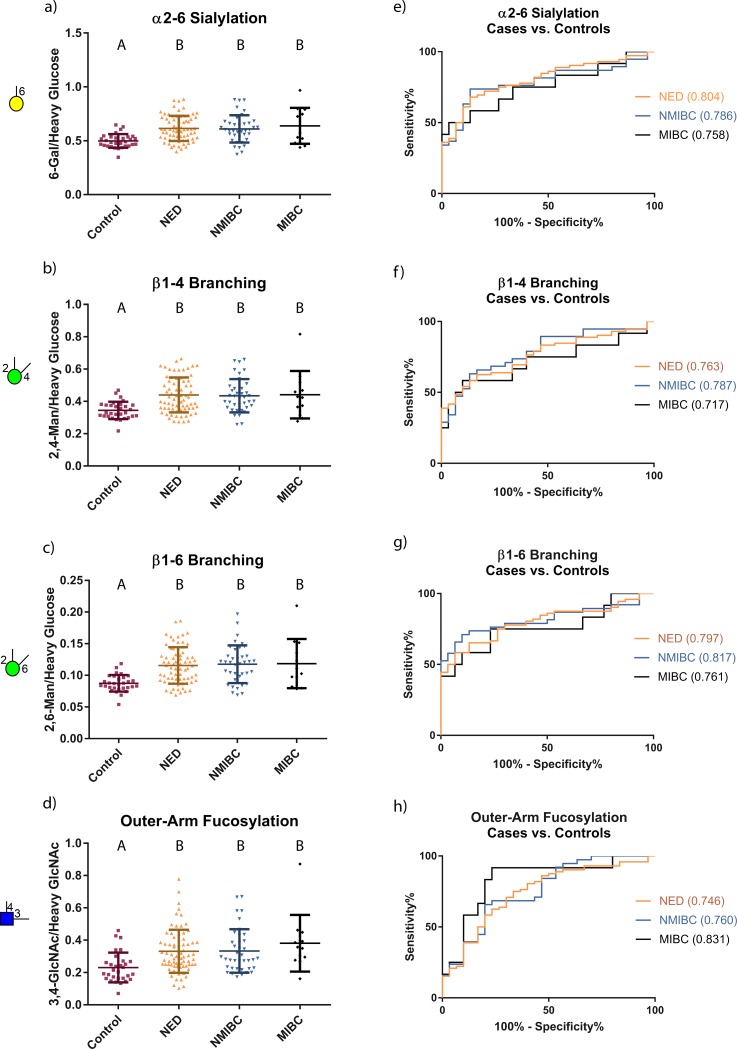
Distributions and ROC curves for the most highly elevated glycan node markers in former & current UCC patients relative to healthy controls when data were normalized to heavy glucose or heavy GlcNAc. Patient distributions are shown in (a-d). The Kruskal-Wallis test was performed followed by Dunn’s post hoc test. The letters at the top of the data points show statistically significant differences between the patient groups; groups with same letter do not have a significant difference. (e-h) ROC curves for the different sub-cohorts of UCC patients vs. healthy individuals. Areas under the ROC curves are provided in parenthesis next to the stated patient groups. As explained in the Discussion, despite the promising AUCs and shapes of some of these ROC curves, these data do not indicate that plasma/serum glycan nodes will potentially serve as clinically useful diagnostic markers of UCC.

**Fig 4:**
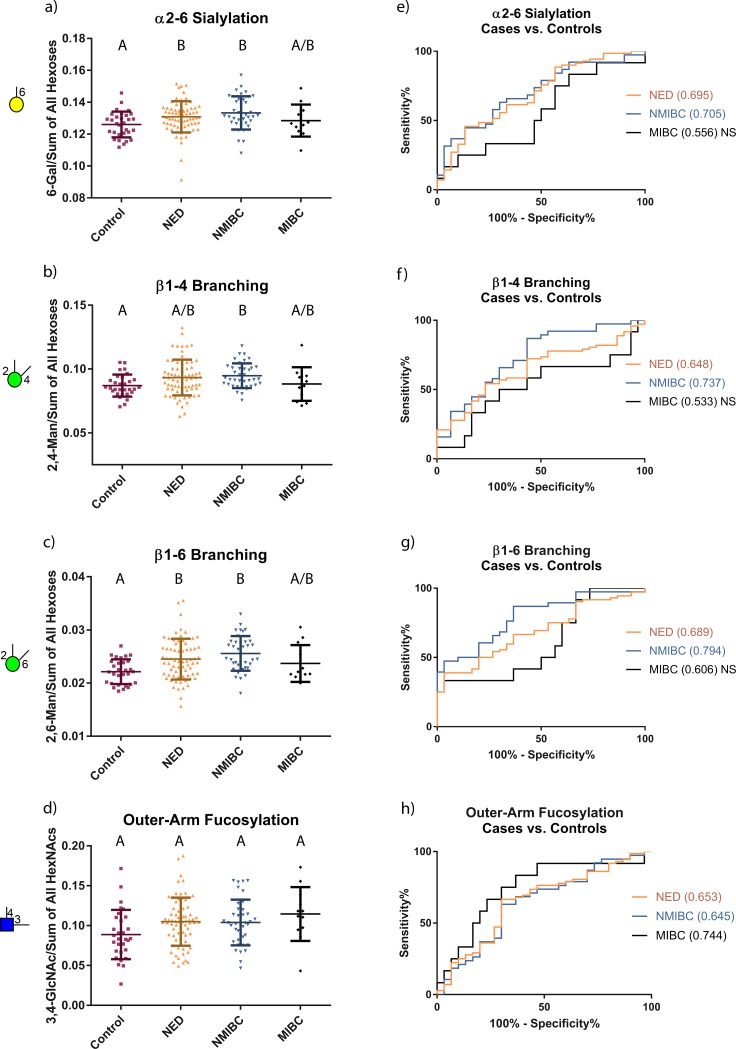
Distributions and ROC curves for the most highly elevated glycan node markers in former & current UCC patients relative to healthy controls when data were normalized to sum of endogenous Hexoses or HexNAcs. Patient distributions are shown in (a-d). The Kruskal-Wallis test was performed followed by Dunn’s post hoc test. The letters at the top of the data points show statistically significant differences between the patient groups; groups with a common letter do not have a significant difference. (e-h) ROC curves for different groups of bladder cancer patients vs. certifiably healthy individuals. Area under the ROC curves are provided in parenthesis next to the stated patient groups. “NS” next to the area under the ROC curves shows that there is no significant difference between the two groups that are being compared. These data do not indicate that plasma/serum glycan nodes will potentially serve as clinically useful diagnostic markers of UCC.

There were four glycan nodes that were most elevated in the current and former UCC patients relative to the certifiably healthy controls, including 6-linked galactose, 2,4-linked mannose, 2,6-linked mannose, and 3,4-linked GlcNAc. These nodes correspond to α2–6 sialylation, β1–4 branching, β1–6 branching, and outer-arm fucosylation, respectively [18, 21]. The univariate distributions of these four glycan nodes in each of the four clinical groups are shown in Fig 3. ROC curves for current and former UCC patients vs. the certifiably healthy controls are also shown (Fig 3). The distribution of each of these glycan nodes within each cohort shows that patients with no evidence of disease (NED) have similar glycosylation profiles to patients with active disease (NMIBC and MIBC) and that significant increases in these glycan nodes can only be seen when comparing current and former UCC patients to the certifiably healthy controls—but not when comparing amongst the three current and former UCC patient subgroups (Table 1 and Fig 3). Data normalized to the sum of endogenous hexoses or HexNAcs were not as effective at distinguishing the control specimens from those from current or former bladder cancer patients (Table 2 and Fig 4). However, as explained in the Discussion section, these data indicate that significant *qualitative* shifts in glycan composition are observed in current and former UCC patients as opposed to mere increases in the absolute abundance of glycans.

**Table 2:** Statistically significant differences between controls and bladder cancer patient sub-cohorts with data normalization to the sum of all endogenous hexoses or HexNAcs.

Glycan Nodes ^a^^,^^b^	Control vs. NED	Control vs. NMIBC	Control vs. MIBC	NED vs. NMIBC	NED vs. MIBC	NMIBC vs MIBC
**t-Fucose**	ns	ns	ns	ns	ns	ns
**t-Gal**	dd	dd	ns	ns	ns	ns
**2-Man**	ns	ns	ns	ns	ns	ns
**4-Glc**	ddd	dddd	d	ns	ns	ns
**3-Gal**	ns	ns	ns	ns	ns	ns
**6-Gal**	i	ii	ns	ns	ns	ns
**3,4-Gal**	ns	ns	ns	ns	ns	ns
**2,4-Man**	ns	ns	ns	ns	ns	ns
**2,6-Man**	ii	iii	ns	ns	ns	ns
**3,6-Man**	ns	ns	ns	ns	ns	ns
**3,6-Gal**	ns	ns	ns	ns	ns	ns
**3,4,6-Man**	ns	ns	ns	ns	ns	ns
**t-GlcNAc**	ns	ns	ns	ns	ns	ns
**4-GlcNAc**	ns	ns	ns	ns	ns	ns
**3-GlcNAc**	ns	ns	ns	ns	ns	ns
**3-GalNAc**	dd	d	ns	ns	ns	ns
**3,4-GlcNAc**	i	ns	i	ns	ns	ns
**4,6-GlcNAc**	ns	dd	ns	ns	ns	ns
**3,6-GalNAc**	ns	ns	ns	ns	ns	ns

^a^ Significance was determined by the Kruskal-Wallis test followed by the Benjamini-Hochberg correction procedure at a 5% false discovery rate.

^b^ “ns” stands for “not significant”. “i” and “d” stand for “increased” or “decreased” glycan levels in the cohort with clinically more advanced disease listed in the column header. “i” or “d” indicates *p* < 0.05, “ii” or “dd” indicates *p* < 0.01, “iii” or “ddd” indicates *p* < 0.001, and “iiii” or “dddd” indicates *p* < 0.0001.

**Fig 5:**
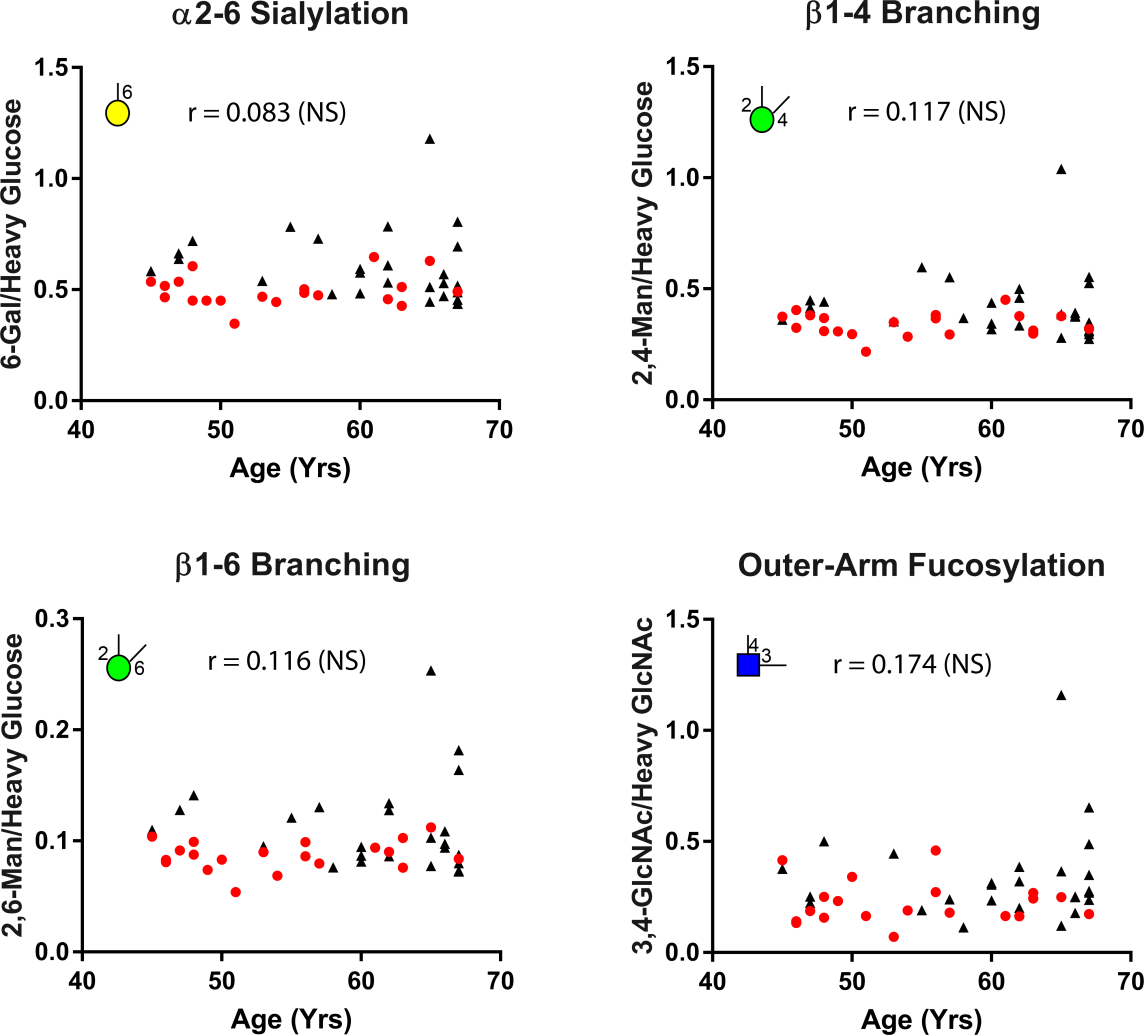
Correlation between age and the most highly elevated glycan node markers in former & current UCC patients relative to healthy controls when data were normalized to heavy glucose or heavy GlcNAc. Pearson correlation was used to evaluate this correlation. The common age range between all cohorts was 45–67. “NS” next to the r-value indicates that the Pearson correlation was not statistically significant. Distribution of the healthy controls is demonstrated by red dots. Distribution of the different sub-cohorts of UCC patients is demonstrated by black triangles.

The average age of the certifiably healthy living kidney donors (controls) was 47, while the average age for the NED, NMIBC and MIBC patients was 74, 76, and 73, respectively. Yet after correcting for multiple comparisons, no statistically significant correlation of any glycan node with age could be found when pooling data from all cohorts and evaluating correlations for the age range in which there was overlap between the controls and the current and former UCC patients (i.e., ages 45–67; see Fig 5). Likewise, no significant correlations with age were observed within the certifiably healthy controls or within the current/former UCC patients when these groups were considered in isolation (not shown).

### Prognostic value of glycan nodes

**Fig 6:**
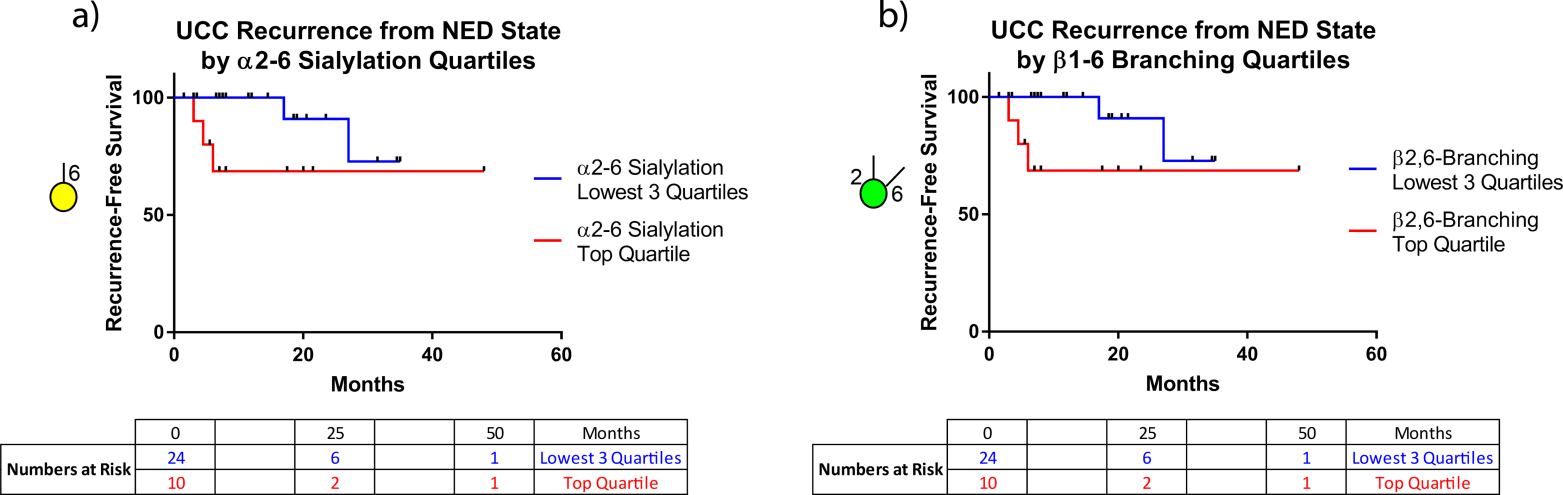
**Bladder cancer recurrence curves** for: (a) The top α2–6 sialylation quartile compared to all other quartiles combined. (b) The top β1–6 branching quartile compared to all other quartiles combined. In both panels, the recurrence curves within each plot were significantly different (log-rank Mantel-Cox test; *p* < 0.05). The median duration of follow-up for those that relapsed, until relapse was 6 months, and for those that did not relapse was 12 months (The median total follow-up time was 11.75 months). The results of Cox proportional hazards models are reported in the Results section.

Within the NED cohort there were numerous samples with high levels of specific glycan nodes that were well out of the range observed in the controls—and which were similar to the cancer patient samples—even though the NED patients were clinically free of disease (Fig 3). These observations led to evaluation of the ability of glycan nodes to predict recurrence in a Cox proportional hazards regression model. After breaking down glycan node data into quartiles and adjusting for age, gender, and time *from* cancer (i.e., time elapsed since there was evidence of cancer in a NED patient), 6-linked galactose and 2,6-linked mannose, which correspond to α2–6 sialylation and β1–6 branching, respectively, predicted recurrence with p-values of < 0.05. The top α2–6 sialylation quartile predicted recurrence from the NED state with a hazard ratio of 15 relative to all other quartiles combined (lower bound at 95% CL = 1.3; upper bound at 95% CL = 180; *p* = 0.029). Similarly, the top β1–6 branching quartile predicted recurrence from the NED state with a hazard ratio of 11 relative to all other quartiles combined (lower bound at 95% CL = 1.2; upper bound at 95% CL = 110; *p* = 0.037). The differences in the rates of recurrence for the top α2–6 sialylation and β1–6 branching quartiles compared to all other quartiles are shown in the progression-free survival curves (Fig 6).

### CRP correlation with glycan nodes

**Fig 7:**
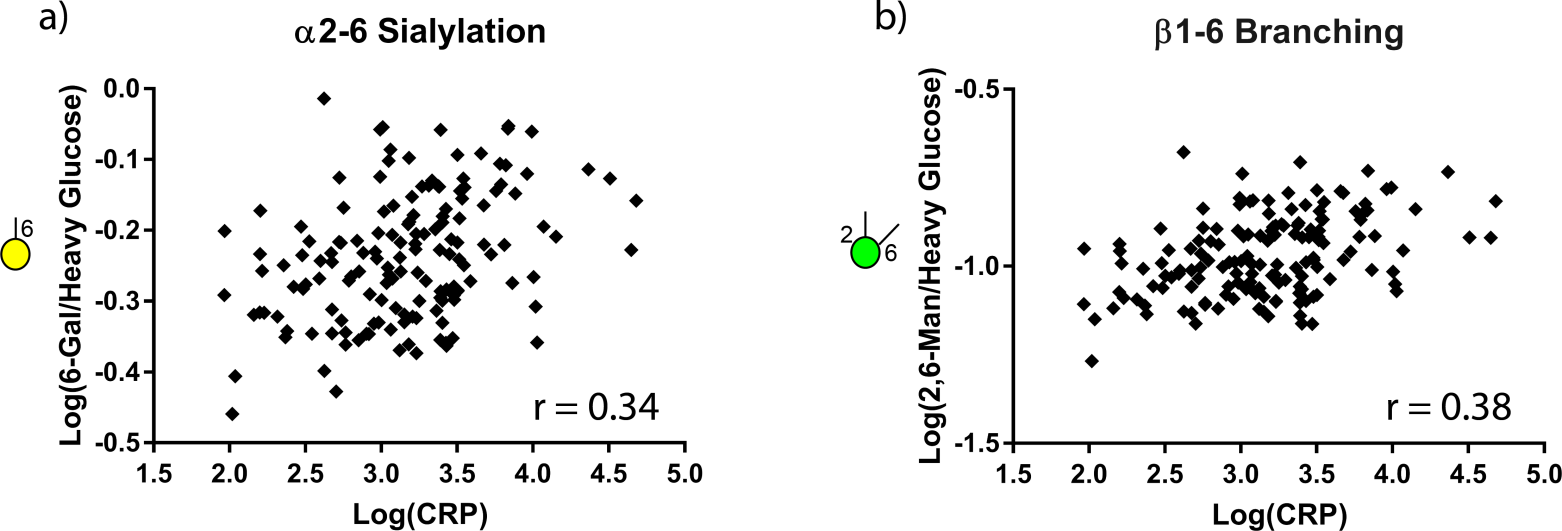
Correlation of CRP and glycan nodes. Log of CRP concentration vs. (a) α2–6 sialylation; r = 0.34 and (b) β1–6 branching; r = 0.38 are plotted. Both correlations are statistically significant (Pearson correlation; *p* < 0.001).

CRP was measured in order to correlate changes in patient glycan nodes with patient inflammation status. The average level of CRP in the certifiably healthy controls was 1.76 mg/L whereas the NED, NMIBC, and MIBC samples had average CRP levels of 3.84, 3.21, and 3.08 mg/L, respectively (which are above the normal range of CRP (<3.0 mg/L) [28]). The levels of 6-linked galactose, which corresponds to α2–6 sialylation, positively correlated with CRP (r = 0.34, p < 0.001), as did the levels of 2,6-linked mannose, which corresponds to β1–6 branching (r = 0.38, p <0.001) (Fig 7).

## Discussion

Out of 19 quantified glycan nodes, four of them, each corresponding to a unique glycan feature including α2–6 sialylation, β1–4 branching, β1–6 branching and outer-arm fucosylation, were most significantly elevated in UCC patients compared to certifiably healthy individuals (Table 1 and Fig 3). Unexpectedly, cancer-free patients with a history of UCC (NEDs) had glycan node distributions that were similar to both the early and late-stage cancer patients but distinct from the controls (Fig 3). And, unlike other types of cancers that we have reported upon previously [21], glycan node-based features were at the same level in later stages of UCC (MIBC patients) as in earlier stages (NMIBC patients). These findings were unanticipated and indicate that the distinct plasma glycan features such as α2–6 sialylation, β1–4 branching, β1–6 branching, bisecting GlcNAc and core fucosylation that were directly quantified by glycan node analysis are not capable of distinguishing patients with active UCC from patients in remission.

In order to interpret the physiological significance of these findings, it must be understood that the glycans being measured are from high-concentration glycoproteins derived primarily from the liver (i.e., transferrin, alpha-2-macroglobulin, haptoglobin, etc) and the immune system (i.e., IgG antibody glycans) rather than being sloughed off or secreted by cancer cells themselves [31, 32]. These macro-level (mg/mL scale) changes in blood plasma glycan biochemistry are thought to be mediated, at least in part, by cytokines secreted from the tumor which are recognized by the liver and/or immune system as part of a systemic inflammatory response, altering the way that these two major glycoprotein-producing systems glycosylate their proteins [33–38].

With this in mind, there are three possible causes for the increases in various glycan nodes observed in Table 1 and Fig 3. First, the acute phase response in current and former UCC patients (evidenced by elevated CRP) may induce a net increase in the total concentration of plasma glycoproteins—and more glycoproteins means more glycans. Second, glycoprotein site occupancy may increase. While this possibility has not been extensively studied, some evidence exists that subtle but statistically significant increases in site occupancy may occur in steatosis and non-alcoholic steatohepatitis [39]. Third, the qualitative nature of the glycans themselves may change. This phenomenon has repeatedly been documented in cancer and is often the primary reason for shifts in glycan profiles—particularly when the data reported are compositional in nature (i.e., all signals sum to 100%) [23, 40, 41]. When the glycan node data from this study are normalized to the sum of endogenous hexose residues or HexNAc residues, statistically significant increases in the four most elevated glycan nodes are observed in current and former UCC patients relative to the healthy controls (Fig 4)—though these increases tend not to be as strong as when total glycan node quantities are considered (Fig 3)—i.e., when the data are normalized to heavy labeled internal standards. Altogether, elevated CRP levels and the data seen in Fig 4 suggest that both the first and the third possible explanations likely contribute to our observations. Assessing changes in glycan site occupancy requires establishing a complex, custom assay for each protein in question and is beyond the scope of the present study.

**Fig 8:**
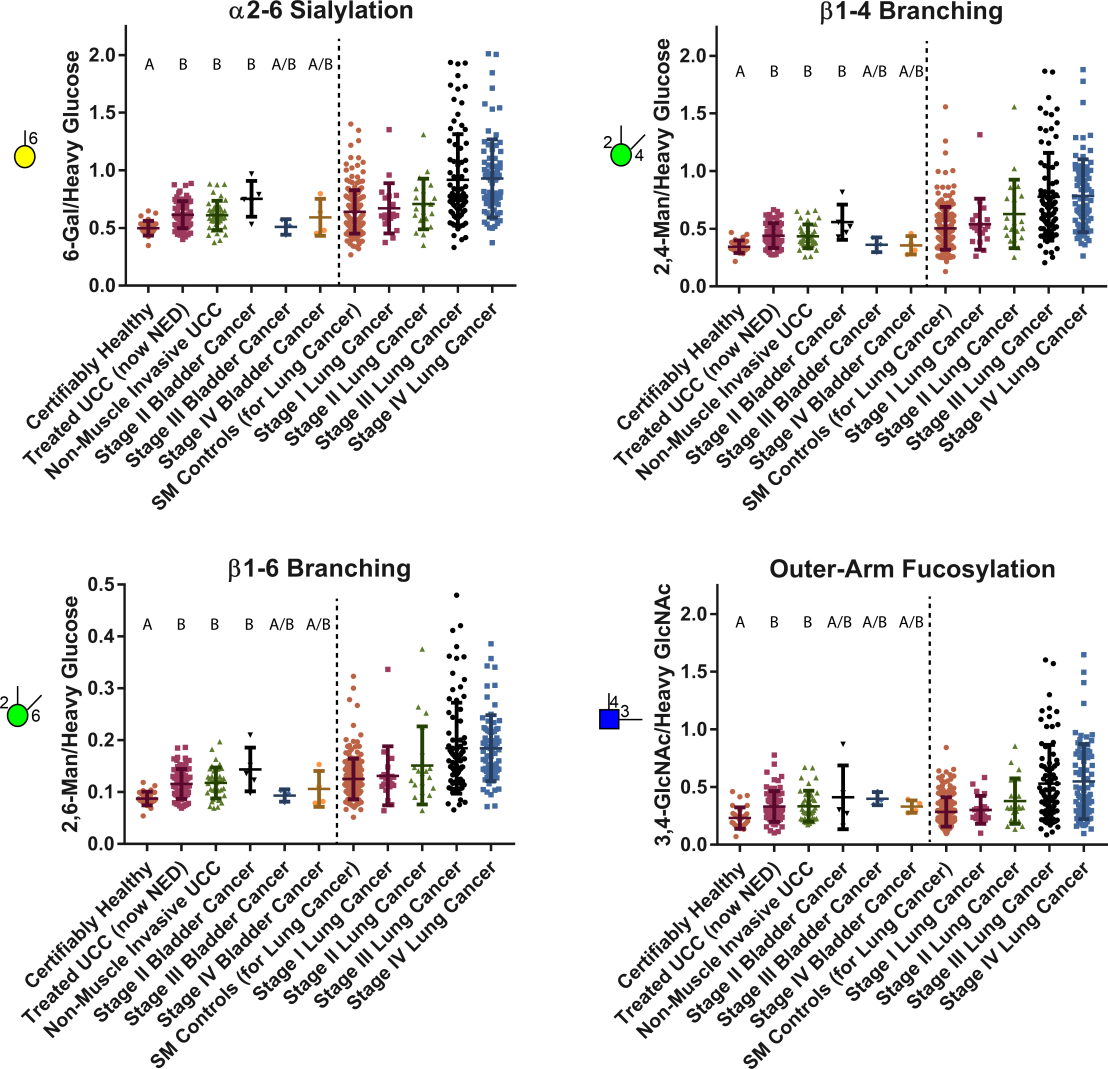
Distribution of the most highly elevated glycan node markers in former & current UCC patients relative to healthy controls with the MIBC group separated by patient stage. Data from our recently published lung cancer study [21] are displayed side-by-side for qualitative comparison. “SM Controls” indicates smoking status matched to the lung cancer patients on the basis of “current”, “former”, or “never-”smoker status. Letters at the top of each cohort show statistically significant differences between the patient groups; groups with a common letter do not have a significant difference.

Overall, the glycan node distributions observed here in UCC suggest that UCC makes modest, early-stage alterations to blood plasma glycans that, even at stages III-IV, do not reach the extreme levels observed in pancreatic, ovarian, lung and other types of cancer [21]. To illustrate, lung cancer patient glycan node data from our recently published article [21] are compared side-by-side with UCC patient glycan node data in Fig 8. It is notable that glycan nodes from the smoking-matched (SM) cancer-free controls from this lung cancer study are quite similar in their overall distributions to the NED, NMIBC and MIBC patients in the present UCC study—and yet are strikingly elevated above the certifiably healthy controls [21]. As we previously described [21], smoking status (provided as “never-“, “former-”or “current smoker”) within this control group had a minor but statistically significant impact on outer-arm and total fucosylation as well as β1–6 branching, but not on α2–6 sialylation or β1–4 branching. Yet smoking alone did not completely account for the elevation of glycan nodes in this control cohort relative to the certifiably healthy controls. Correspondingly, even in remission, most former UCC patients with no evidence of disease (NED), tended to maintain modestly elevated blood plasma glycan levels—wherein the NED patients with the most highly elevated levels were most likely to experience relapse (Fig 6).

Though the reason(s) for glycan node elevation in nominally cancer-free individuals are not fully known, it has previously been shown that serum glycans can be elevated in inflammatory patient states in the absence of cancer [22–24]. Moreover, chronic inflammation is known to be closely associated with the development of cancer [42–44]. Together with the observations presented here, this suggests that the elevated plasma glycan levels observed in former UCC patients (currently in the NED state) that are prognostic of recurrence may be driven by or simply part of inflammatory processes. To assess this possibility, we measured CRP concentrations and found them to be strongly significantly correlated with levels of both α2–6 sialylation and β1–6 branching (Fig 7)—an observation that goes hand-in-hand with the fact that CRP has been found to predict UCC patient survival [26, 28].

This brings up the question of whether or not there is a mechanistic connection between alterations in plasma glycans (associated with inflammation) and the development or progression of cancer. There is evidence for the concept that the biological landscape experiences “grooming” or premetastatic “niche” formation prior to cancer establishing residence within the body [45–49]. And while glycans are not solely responsible for this process, evidence exists that they play important roles. As we have previously summarized [21] and others have explained in detail, cell-surface glycans that facilitate resistance of galectin-mediated apoptosis [48, 50–52] (including poly-N-acetyllactosamine modified core 2 O-glycans [53–58]) as well as sialylated glycans that stimulate the inhibitory Siglec-7 receptor on natural killer cells [53–56] have important roles to play in helping cancer evade the body’s natural immunity.

Using glycan node analysis, we have previously observed major changes in distinct P/S glycan features in lung, pancreatic, ovarian [21], and breast cancers [20]. The results presented here show that, relative to healthy individuals, there is a significant alteration of P/S glycan features that correlates with inflammation and is present at the onset of UCC—but that, unlike other types of cancer that we have observed to date [21], does not change in a stage-dependent manner—even when UCC patients go into remission. Certifiably healthy individuals cannot be considered to be clinically relevant controls for the development of cancer diagnostics—but they do illustrate the striking changes in blood biochemistry that occur as cancer develops and takes hold in the human body. Thus taken together with our previous observations in lung cancer [21], the findings presented here suggest that if there are clinical applications for P/S glycan node measurements, they most likely lie in evaluating cancer patient relapse or progression risk—or in monitoring nominally healthy persons who exhibit behaviors such as smoking that put them at risk for the biochemical transition between a genuinely healthy state and one in which their blood chemistry (above and beyond mere behavior) reveals a truly high-risk state. Ultimately, however, further study is required to elucidate the potential mechanistic role of these macro-level changes to blood glycan biochemistry in the development and overall progression of cancer.

## Conclusions

α2–6 sialylation, β1–4 branching, β1–6 branching, and outer-arm fucosylation were found to be significantly elevated in both current and former (in remission) UCC patients relative to certifiably healthy living kidney donors, with ROC curve c-statistics averaging approximately 0.8—yet this does not make them clinically relevant diagnostic biomarkers of UCC. In contrast to the stage-dependence that we have observed in other types of cancer [21], differences between patients with muscle invasive UCC, non-muscle invasive UCC and patients in remission were not statistically significant. For UCC patients in remission, α2–6 sialylation and β1–6 branching were prognostic indicators of recurrence and were correlated with CRP levels (r = 0.34 & 0.38, resp.; p < 0.001), a known prognostic marker in UCC. Though glycan nodes exhibited less stage-dependency in UCC than in other cancers [21], results highlighted the pronounced difference between the serum glycan biochemistry of healthy individuals vs. any stage of UCC (including remission) and underscored the concept (previously observed [21]) that for plasma glycans the transition between a healthy state and an at-risk state is much more pronounced than that between an at-risk state and early stage cancer.

## Supporting information

S1 Appendix Inclusion and Exclusion criteria for certifiably healthy living kidney donors. (PDF)

S1 File Extracted-ion chromatographic peak areas for all samples, including their normalization to heavy glucose or heavy GlcNAc and normalization to the sum of all endogenous hexoses or HexNAcs as well as %CV values for batch-to-batch QC samples. (XLSX)
