# Supplementary material for: Behavior of blood plasma glycan features in bladder cancer
Source: PLoS One. 2018 Jul 24;13(7):e0201208. doi: 10.1371/journal.pone.0201208 (PMC6057681; doi:10.1371/journal.pone.0201208)
Supplement: S1 Appendix — (PDF) [file pone.0201208.s001.pdf]

<u>Inclusion and Exclusion Criteria for Certifiably Healthy Living Kidney Donors</u>

Mayo Clinic Arizona Guidelines

Inclusion Criteria

All persons that wish to be considered as a potential living donor will be evaluated using the following criteria:

- Age
  - Between the ages of 18-70
  - Those above age 70 will be considered on a case-by-case basis
- BMI and Blood Glucose
  - Evaluation of Living Kidney Donor: BMI and Glucose Metabolism Guideline
    - Impaired Fasting Glucose (FBS > 100 and < 126) or IGT (2 hour BS > 140 and < 200) is a relative contraindication to donation. Patients with family history of diabetes, gestational diabetes and/or metabolic syndrome may be at higher risk than others for developing DM.
    - Prospective donors meeting these criteria require counseling about increased risk of developing DM and its consequences.
- Blood Pressure
  - All patients worked up at Mayo Clinic will complete either a 24 hour blood pressure monitor, a blood pressure taken on at least two different occasions, or overnight BP monitoring to be analyzed
  - Criteria for the diagnosis of Hypertension
    - Clinic or hypertensive therapy nurse blood pressure > 140/90 mm Hg
    - ABPM awake period (mean value) > 135/85 mm Hg
    - ABPM overall (mean value) > 130/80
  - Donor Selection
    - Normal Blood pressure acceptable as a donor
    - Hypertension may be acceptable if all met:
      1. Greater than age 40
      2. Caucasian

3. GFR meets Evaluation of Living Kidney Donor: GFR Protocol

4. Hypertension controlled with one drug + diuretic

- Kidney Function – GFR Standards
  - Evaluation of Living Kidney Donor: GFR Protocol
- Malignancy
  - Evaluation of the Living Kidney Donor: Donor Malignancy Guideline
- Crossmatch/ABO
  - Arizona: See Living Donors Blood Type, Subtype Determination Policy and ABO Verification for Living Donors Policy
  - Florida: See Living Donors Blood Type, Subtype Determination Policy and See ABO Verification for Living Donors Policy
  - Rochester: See ABO Blood Group and Other Vital Data Compatibility Verification Guideline
- Pulmonary Nodules
  - Evaluation of the Living Kidney Donor: Pulmonary Nodules Guideline
- Stones
  - Evaluation of the Living Kidney Donor: Donor Nephrolithiasis Guideline
- Microscopic Hematuria
  - Evaluation of the Living Kidney Donor: Donor with Microscopic Hematuria Guideline
- Polycystic Kidney Disease
  - Evaluation of the Living Donor: Polycystic Kidney Disease Guideline
- Psychiatric
  - Evaluation of the Living Donor: Psychiatric Evaluation policy
- Donor Coercion
  - Evaluation of the Living Donor: Coercion Guideline

Exclusion Criteria

- The transplant center may exclude a donor with any condition that, in the hospital's medical/ethical judgment, causes the donor to be unsuitable for organ donation.

- The transplant center will exclude all donors who meet any of the following exclusion criteria:
    - Is less than 18 years old
    - Is mentally incapable of making an informed decision
    - History of HIV
    - Infectious Disease that can be transmitted through transplantation
    - Active malignancy, or incompletely treated malignancy
    - High suspicion of donor coercion
    - High suspicion of illegal financial exchange between donor and recipient
    - Evidence of acute symptomatic infection (until resolved)
    - Uncontrolled diagnosable psychiatric conditions requiring treatment before donation, including any evidence of suicidality
    - Uncontrollable hypertension or history of hypertension with evidence of end organ damage
    - Diabetes mellitus
    - Consider on an individual basis, usually not accepted as donor
        - Non-Caucasian with hypertension
        - Other antihypertensive regimens
        - Family history of hypertensive kidney injury
        - Evidence of end organ damage such as Left Ventricular Hypertrophy (LVH)
        - Additional risk factors particularly active smoking

Additional Information Regarding Living Kidney Donor Selection

Donor Screening

- A potential donor will be screened by the living donor coordinator on the phone or by completing an electronic form.
- In addition, a social work interview is to be conducted for all potential donors if requested by the living donor coordinator.

- At that point, blood type and tissue typing will be obtained and reviewed by the donor team.
- If suitable, potential donor will be scheduled for an evaluation.

Donor Evaluation

Living donor candidate workups are valid for a duration of 18 months after being accepted at selection conference; thereafter, any repeat testing necessary will be determined by the evaluating team at the donor site after which the candidate will again be presented at selection conference.

Workup of the donor will include:

- Labs:
  - CBC with differential
  - PT/INR and PTT
  - Renal Profile (BUN, Creatinine, Electrolytes)
  - Fasting glucose and A1C
  - Liver function profile
  - Thyroid Stimulating Hormone
  - Fasting Lipid Profile
  - Serum protein electrophoresis for age >60
  - Oral glucose tolerance test for high risk patients per the Evaluation of Living Kidney Donor: BMI and Glucose Metabolism Guideline
  - HCG Quantitative blood (female < 55)
- Serologies:
  - See Infectious Disease Protocol
- Clearance Studies and 24 hour urine collection:
  - Spot micro albumin/creatinine ratio
  - Iothalamate GFR clearance and 24 hour creatinine clearance
- Urine tests:
  - Routine urinalysis
  - Midstream Gram stain and culture

    - Urine microscopy
    - Stone risk profile for any donor with history of nephrolithiasis per the Evaluation of Living Kidney Donor: Donor Nephrolithiasis Guideline
- Other tests:
    - Chest x-ray
    - EKG
    - Exercise Stress Echo (> 60 years or high cardiac risk) (>50 with hypertension or tobacco use) and/or dobutamine or nuclear stress test if clinically appropriate (may consider for younger patients on case to case basis)
    - Screening for Autosomal Dominant Polycystic Kidney Disease (ADPKD) (for related donors of ADPKD recipient)
        - Per the Evaluation of Living Kidney Donor: Polycystic Disease Guideline
    - CT angiography: renal protocol to determine:
        - Whether the kidneys are of equal size
        - If the kidneys have masses, cysts, or stones
        - If the kidneys have other anatomical defects
        - Which kidney is more anatomically suited for transplant
- Cancer screening per American Cancer Society (ACS) guidelines:
    - Mammogram (females ≥40 or if h/o breast cancer in pre-menopausal 1st degree relative)
    - Cologuard (≥50 or family history) - first tier for low-risk patients
    - Females should have a Pap smear every three years, provided most recent Pap smear was normal. If most recent Pap smear was not normal, follow-up should be according to the recommendations of the GYN service.
    - PSA
        - Age 50 for men who are at average risk of prostate cancer and are expected to live at least 10 more years
        - Age 45 for men at high risk of developing prostate cancer. This includes African Americans and men who have a first-degree relative (father, brother, or son) diagnosed with prostate cancer at an early age (younger than age 65)

        - Age 40 for men at even higher risk (those with more than one first-degree relative who had prostate cancer at an early age)
    - Low-Dose CT scan for those at high risk for lung cancer (those who meet all of the below):
        - 55 to 74 years of age
        - Have at least a 30 pack-year smoking history AND are either:
            - Still smoking OR
            - Have quit within the last 15 years

Note: A pack-year is the number of cigarette packs smoked each day multiplied by the number of years a person has smoked. Someone who smoked a pack of cigarettes per day for 30 years has a 30 pack-year smoking history, as does someone who smoked 2 packs a day for 15 years.

- Consultations:
    - Nephrology (different physician from recipient if possible)
    - Living Donor Nurse Coordinator
    - Social Services (different from recipient social worker if possible)
    - Nutrition
    - Pharmacy
    - Transplant Surgeon/Urologist
    - Consider Transplant Psychiatrist/Clinical Psychology Specialist
    - Independent Living Donor Advocate
- Any additional tests, procedures, consults or biopsies needed to determine their candidacy is a part of the donor evaluation until the donor is ruled out as a donor

Selection Conference

- The final decision to proceed with donation made at the Selection Conference, which may include the following multi-disciplinary team members: transplant surgeons, nephrologists, independent living donor advocate, psychiatrist, social worker, dietitian, pharmacists, financial services and nursing personnel.
- Cases will be presented to the Selection Committee once all appointments completed and all results available.

- Results of testing and assessments will be reviewed by the multi-disciplinary Selection Committee, providing an opportunity for all members to raise concerns and discuss any issues regarding the donor's suitability.
- The living donor's suitability for donation will be thoroughly documented in the donor's medical record
- The decision of the committee will be documented in the donor's medical record and communicated to potential donor by a member of the multidisciplinary team.
- Any exceptions to the selection criteria must be approved by the Living Kidney Donor Selection Committee & the reasons for it thoroughly documented in the patient's medical record
